# Gastrostomy for Malignant Stricture of the Æsophagus. Hydatid Cyst of Liver

**Published:** 1883-07

**Authors:** 


### GASTROSTOMY FOR MALIGNANT STRICTURE OF THE ÆSOPHAGUS. HYDATID CYST OF LIVER. By the Editor.

R. C., aged 53, a shopkeeper, was admitted to the Bristol Infirmary, under the care of Dr. Shingleton Smith, on October 6th, 1881, complaining of difficulty in swallowing food, great weakness, shortness of breath on exertion, and swelling of the ankles and legs.

His *history* was fairly good. There was no predisposition to hereditary complaints, and no evidence of syphilis. He had been a butler and accustomed to partake freely of stimulants, but seldom to great excess. He had had two or three mild attacks of gout, and three years previously he had dropsy of the lower extremities. Seven years ago he had occasional attacks of violent pain in the pit of the stomach, followed by profuse vomiting. With these exceptions he had enjoyed very good health.

He first noticed that he could not easily swallow solids two months before admission. For a few weeks prior to admission he had lived entirely on liquid food, being unable to swallow the smallest bits of solid material. He had got very thin and weak, and his ankles had become much swollen.

*On admission* his symptoms were chiefly those of want of sufficient nourishment. He looked pale, thin and haggard, had great shortness of breath on walking even a few yards, and complained of a constant feeling of nausea which was much aggravated when he attempted to take food. Both ankles and legs were œdematous. There was a systolic murmur at the apex of the heart, but no cardiac enlargement. Pulse weak, 60–70, occasionally intermitting. Arteries tortuous and hard; slight arcus senilis. The cutaneous veins on the chest, especially on the left side, were enlarged and full of dark blood. Urine was of normal specific gravity and reaction, no albumen. Weight, 9st. 8lb. He was put on iodide of potassium in increasing doses up to gr. 45 three times a day. In three weeks he had lost ten pounds in weight, and though his power of swallowing was improved, yet he was clearly losing ground.

On October 30th I was asked to see him. With some difficulty a No. 12 French catheter was passed into the stomach. The catheter was firmly gripped and appeared to traverse a stricture of some length. No tumour could be felt with the fingers, but the obstruction did not begin less than an inch below the larynx. The mucus in the eye of the catheter was examined microscopically, but no information was derived therefrom. Next day he had severe pain shooting up from the right side of the neck to the right eye and temple, and he said his right ear felt numbed. The difficulty in swallowing was also increased.

The pros and cons of the operation of gastrostomy were honestly put before him and he was left to elect for himself. In the meantime nutrient enemata were administered twice daily. He decided not to be operated upon, and left the Infirmary on November 19th.

On December 1st he appeared for re-admission saying he had got much worse and requesting operation, and he then came under my care. He was then in the last stage of exhaustion, weighing only 8st. 1lb., unable to support himself standing and looking very haggard. His legs were more œdematous, and the mitral murmur was somewhat more marked. Urine sp. gr. 1005, acid, no albumen.

He was put on nourishing liquid diet and enemata in preparation for operation, and in four days gained four and a half pounds in weight. The day before the operation his bowels were well moved by enema, and no other preparatory treatment was adopted.

The *operation* was performed antiseptically on December 13th. An incision an inch and a half in length along the edges of the eleventh and twelfth ribs and about a finger's breadth distance from them. On dividing the peritoneum the liver filled the wound, and went for nearly two inches to the left of it. A retractor was put under its thin margin and it was kept to one side during the rest of the operation by an assistant. (The cause of the liver being pushed so much to the left side was found after death to be the presence of a large hydatid cyst on the upper surface of the right lobe). The stomach was found high up under the diaphragm contracted to a size not larger than the colon, very thin walled and quite empty. Some slight amount of force had to be used to get a portion of its wall into the wound. The portion of stomach selected for opening had reference rather to the least amount of dragging on its walls than to the best position physiologically, and this was found to be at a point nearer the lower than the upper margin and about three inches distant from the pylorus. The wall of the stomach was fixed to the margins of the wound by a thick continuous silver wire carried round each margin in the manner in which shoemakers stitch the sole to the upper of boots. It included skin, peritoneum and fibrous coat of stomach, and when the wires were pulled almost straight exact apposition of peritoneal surfaces was got. It was at first intended to use interrupted sutures, but it was clear that the traction on the stomach walls would try too much the small grip which they would afford and also probably leave little openings between the peritoneal surfaces, and so the above plan was adopted. It was not easy of application, but was most efficient. An area of stomach wall about three-quarters of an inch long and half an inch broad appeared in the bottom of the wound.

The patient did very well after operation. The temperature on the morning after operation was 99.4, thereafter it continued normal till the seventh day. No sickness. The patient was fed on nutrient enemata of peptonised milk and beef juice and Slinger's capsules. On the evening of December 16th he began to regurgitate quantities of most offensive mucus, and this continued on the 17th. On this day the antiseptic dressings were removed and the stomach opened with a Syme's knife. A French gum elastic catheter was placed in the opening and three ounces of warm peptonised milk were introduced, aud this was repeated every four hours till next day. He then felt much better, and all regurgitation had ceased. His breath did not smell so offensively. Next day he had gained no ground however, and thereafter it became evident that his food did him no good, and he gradually sank, dying on the 23rd, ten days after operation.

At the *post mortem* examination the stomach was found well glued to the peritoneum round the incision by recent lymph, and the rest of the peritoneum was quite free from signs of inflammation. The aperture in the stomach was nearer to the pyloric than to the cardiac end of the stomach. There was found (what was not diagnosed during life) a globular hydatid cyst four inches in diameter, with thick walls, embedded in the upper portion of the right lobe of the liver, between it and the diaphragm. When the cyst was enucleated the right lobe was found to be atrophied and the left to be hypertrophied, so that the former weighed only 1lb. 3oz. and the left exactly double,—2lbs. 6oz. The margin of the left lobe was very thin and much spread out, and this was the reason why the liver was found to be so much in the way during the operation. And, in fact, part of the liver margin was embedded in the area of lymph exudation which surrounded the site of operation, and would, no doubt, have become permanently fixed there. The stricture in the æsophagus was of typical epitheliomatous growth, about an inch long, tortuous, and in no part of larger calibre than a No. 6 catheter. The bowels were full of undigested food. All the other organs were healthy.

*Remarks.*—This is one more case in which the operation was too long delayed. It was only at the urgent desire of the patient that it was performed at all. Though it was done almost as a forlorn hope, yet it showed with how slight constitutional disturbance this operation may be performed even when the patient is in the last stage of exhaustion. The presence of a hydatid cyst in the liver, pushing it over to the left side and causing enlargement of the left lobe of the liver and so rendering the operation more difficult than usual, was a most unusual and quite unexpected complication.

